# Clinical significance of CSF3R, SRSF2 and SETBP1 mutations in chronic neutrophilic leukemia and chronic myelomonocytic leukemia

**DOI:** 10.18632/oncotarget.15355

**Published:** 2017-02-15

**Authors:** Yuan Ouyang, Chun Qiao, Yu Chen, Su-Jiang Zhang

**Affiliations:** ^1^ Department of Hematology, Ruijin Hospital North Shanghai Jiao Tong University School of Medicine, Shanghai 200000, China; ^2^ Department of Hematology, The First Affiliated Hospital of Nanjing Medical University, Jiangsu Province Hospital, Collaborative Innovation Center For Cancer Personalized Medicine, Nanjing Medical University, Nanjing 210029, China

**Keywords:** CNL, CMML, gene mutation, CSF3R, SRSF2

## Abstract

Chronic neutrophilic leukemia (CNL) and chronic myelomonocytic leukemia (CMML) are rare hematologic neoplasms. We performed *CSF3R*, *SRSF2* and *SETBP1* mutational analyses in 10 CNL and 56 CMML patients. In this sample cohort, 80% of CNL patients harbored *CSF3R* mutations, of which the *CSF3R* T618I mutation was dominant. Mutations in *CSF3R* and *SETBP1* were found in 7.1% and 5.3% CMML patients respectively, while 25% of CMML patients carried *SRSF2* mutations. Strikingly, we identified that all of the *CSF3R* mutations detected in CMML patients were represented by a P733T mutation. The *CSF3R* P733T mutation represents a novel *CSF3R* mutation. In addition, none of the four *CSF3R* P733T mutated patients carried *SRSF2* mutations [0/14 (0%) patients with combined *CSF3R* P733T and *SRSF2* mutations *vs*. 4/42 (9.5%) with *CSF3R* P733T and wt *SRSF2*, *P* < 0.001]. Both mut *SRSF2* and mut *SETBP1* patients had shorter overall survival (OS) and progression-free survival (PFS) compared to patients with wt *SRSF2* (*P* < 0.001 both) and wt *SETBP1* (*P* < 0.001 and *P* = 0.02, respectively). While we found no significant differences in OS and PFS as a consequence of *CSF3R* mutation status, our work suggest that the *CSF3R* T618I mutation is a diagnostic marker with good specificity and sensitivity for CNL. In conclusion, our study highlights effective diagnostic and prognostic markers of CNL and CMML patients in the Chinese population.

## INTRODUCTION

CNL is a rare hematologic neoplasm that is diagnosed largely based on exclusion of underlying causes of reactive neutrophilia and/or the lack of specific molecular markers of other hematological malignancies [1]. Until the recent discoveries of *CSF3R* and *SETBP1* mutations [2], no recurrent genetic abnormalities have been identified in CNL.

CSF3R (G-CSF-R), the colony-stimulating factor 3 receptor, is a trans-membrane protein which plays a prominent role in the growth and differentiation of granulocytes [3]. While *CSF3R* mutations are most commonly found in severe congenital neutropenia (SCN), the rates of *CSF3R* mutations rises sharply upon progression to secondary acute myeloid leukemia (sAML) [4–6]. Thus, *CSF3R* mutations may critically influence on disease progression to AML, although the types of truncation mutations that associate with sAML are rarely detected in other disorders, including de novo AML*6*. Recently, acquired *CSF3R* mutations (in particular the *CSF3R* T618I mutation) were described in a majority of patients diagnosed with CNL or atypical chronic myeloid leukemia (aCML) [2, 7]. While the relevance of *CSF3R* mutations in CMML has also been investigated, discrepancies exist among different studies. For example, Kosmider et al. identified about 3% of CMML patients with *CSF3R* somatic mutations, while Pardanani et al failed to identify *CSF3R* mutations in CMML [8, 9].

CMML is the most frequent entity among myeloproliferative/myelodysplastic neoplasms (MDS/MPN) [10]. About 90% of CMML patients carry genomic aberrations, which includes mutations in genes encoding for epigenetic regulators (TET2, ASXL1, DNMT3A, EZH2, IDH1, IDH2), spliceosome components (*SRSF2*, SF3B1, ZRSF2, U2AF1), transcription factors (RUNX1, NPM1, TP53) and other signaling molecules (NRAS, KRAS, CBL, JAK2, FLT3) [11]. Of the many genes found mutated in CMML, *SRSF2* mutations are dominating. *SRSF2* mutations may excert oncogenic activity by regulating alternative splicing through prevention of exon skipping [12]. The mutational frequency of *SRSF2* in MDS, CMML and sAML were reported to be 10–15%, 21–47% and 6.5–24%, respectively [13]. Interestingly, an *SRSF2* mutation was also detected in one CNL patient [14].

Apart from mutations in *CSF3R* and *SRSF2*, mutations in *SETBP1* has also been demonstrated in hematological malignancies. Piazza et al. discovered *SETBP1* mutations in 24% of aCML, 10% of unclassified MDS/MPN, 4% of CMML and in 25% of CNL cases [15]. Likewise, Damm et al. observed a frequency of *SETBP1* mutations of 1.7%, 2.2% and 6.2% in sAML, MDS and CMML, respectively [16].

Here, we have unveiled the frequency, clinical significance and prognostic relevance of mutations in *CSF3R*, *SETBP1* and *SRSF2* in a cohort of 10 CNL and 56 CMML patients, with the aim of providing insights into the development of effective diagnostic and prognostic tools of CNL and CMML patients in the Chinese population.

## RESULTS

### Mutational landscape in the patients

**Figure 1:**
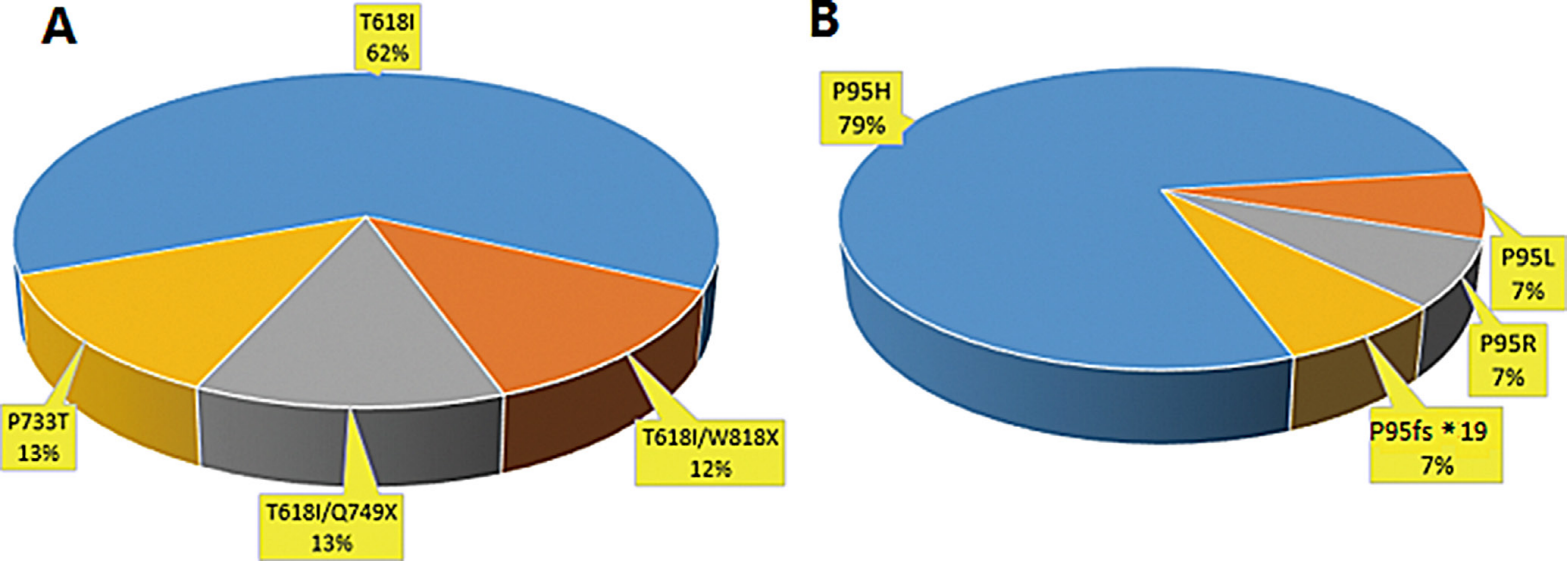
Frequency distribution of *CSF3R* and *SRSF2* mutations in CNL and CMML patients (**A**) In 10 patients with CNL, 8(8/10, 80%) patients had *CSF3R* mutation and 7(7/8, 87.5%) of them were with *CSF3R* T618I. (**B**) In 56 CMML patients, 14(14/56, 25%) patients were found to have *SRSF2* mutations, including P95H, P95L, P95R and P95fs*19.

**Figure 2:**
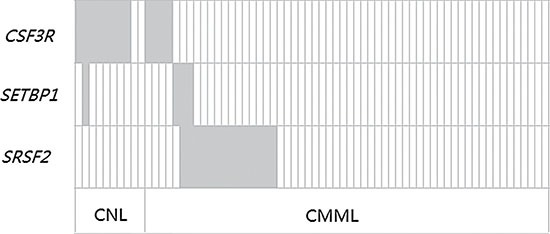
Frequency distribution of *CSF3R*, *SETBP1* and *SRSF2* genetic aberrations in CNL and CMML patients Each box indicates 1 patient. Dark gray boxes are indicative for patients who are positive for the respective mutation; light gray boxes indicate wild type status.

In our study, we found that 80% (8/10) of CNL patients harbored *CSF3R* mutations. Intriguingly, 87.5% (7/8) of patients carried *CSF3R* T618I substitution mutations and 2 carried double mutations (T618I together with W818X and Q749X, respectively). One CNL patient carried a *CSF3R* P733T mutation (Figure 1A). The patient with T618I/Q749X double mutations also had a *SETBP1* D874N mutation. In 56 CMML patients, 25% (14/56) of patients were found to have *SRSF2* mutations (P95H (11 patients), P95L (1 patient), P95R (1 patient) and P95fs*19 (1 patient)) (Figure 1B). In addition, 7.1% (4/56) of CMML patients had a *CSF3R* P733T mutation and 5.3% (3/56) had *SETBP1* mutations (I871T (2 patients) and D868N (1 patients)) (Figure 2). No *CSF3R*, *SRSF2* or *SETBP1* mutations were identified in patients diagnosed with MDS, CEL or in healthy donors (Table 1). All of the gene mutations identified in our study were somatic mutations.

**Table 1:** *CSF3R*, *SETBP1* and *SRSF2* mutational status in different hematological malignancies

Diagnosis	*CSF3R* Mutation (%)	*SETBP1* Mutation (%)	*SRSF2* Mutation (%)
CNL	8/10 (80)	1/10 (10)	0/10
CMML	4/56 (7.1)	3/56 (5.3)	14/56 (25)
CEL	0/10	0/10	0/10
MDS	0/20	0/20	0/20

CNL: chronic neutrophilic leukemia; CMML: chronic myelomonocytic leukemia; CEL: chronic eosinophilic leukemia; MDS: myelodysplastic syndromes.

### Impact of mutation status on clinical and biological characteristics

*CSF3R* mutated CNL patients constituted of 7 males and 1 female. The median age was 39 (range 27 to 92 years). These patients had (45 ± 38.39) × 10^9^/L of mean white blood cell (WBC) count, (40.73 ± 37.41) × 10^9^/L of mean neutrophil count, (87.7 ± 5.53)% of mean neutrophil percentage, (100.25 ± 33.1)g/L of mean hemoglobin(HB) level and (148.13 ± 124.34) × 10^9^/L of mean blood platelet cell (BPC) count. Further, blast cell count of peripheral blood (PB) and bone marrow (BM) were (2.13 ± 4.02)% and (3.63 ± 2.62)%, respectively. One patient with an abnormal karyotype (47, XY, +8) was observed. During a median follow-up of 22 months (range: 4–37 months), 2 *CSF3R* mutated CNL patients died (one case died because of disease evolution into ANLL-M2). Of two wt *CSF3R* CNL patients, an evolution to acute plasma cell leukemia (APCL) was observed in the patient with MGUS-CNL (Table 2). In CMML, none of the 4 *CSF3R* mutated patients were detected with *SRSF2* mutations [0/14(0%) vs. 4/42(9.5%), *P <* 0.001]; 1 of the 3 patients with *SETBP1* mutations also had *SRSF2* mutations [1/14(7.1%) vs. 2/42(4.8%), *P >* 0.05]. Characteristics such as age, gender, WHO category, FAB category, karyotype, blood cell counts and CPSS risk stratification did not reveal any difference between mutated *SRSF2* patients and wt *SRSF2* patients (*P >* 0.05) (Table 3).

**Table 2:** Clinical characteristics and laboratory variables of CNL patients

No.	Age	Sex	Diagnosis	WBC	N%	HB	BPC	PB Blast	BM Blast	Karyotypes	*CSF3R*	*SETBP1*	*SRSF2*	Disease progression	Death
1	33	M	CNL	21.6	94.1	153	115	0	1.2	NK	T618I/ W818X	WT	WT	N	N
2	65	M	CNL	26.9	86.0	76	17	0	3.6	NK	T618I/ Q749X	D874N	WT	N	N
3	27	F	CNL	62.5	80.3	72	17	10	6.4	NK	T618I	WT	WT	Y	Y
4	56	M	CNL	134.5	96.1	118	20	7	8.4	NK	T618I	WT	WT	N	N
5	34	M	CNL	26.5	81.2	74	224	0	3.6	47,XY, +8	T618I	WT	WT	N	N
6	32	M	CNL	28.0	86.8	63	188	0	3.6	NK	T618I	WT	WT	N	N
7	44	M	CNL	39.7	88.6	129	273	0	1.6	NK	T618I	WT	WT	N	Y
8	92	M	CNL	20.6	88.5	117	331	0	0.0	NK	P733T	WT	WT	N	N
9	57	M	CNL	84.8	96.1	81	50	0	1.6	NK	WT	WT	WT	N	N
10	77	M	MGUS- CNL	55.2	90.2	129	227	0	0.0	NK	WT	WT	WT	Y	Y

CNL, Chronic neutrophilic leukemia; MGUS-CNL, undetermined significance monoclonal gammopathy associated with chronic neutrophilic leukemia; WBC, white blood cell; N%, neutrophil percentage; HB, hemoglobin; BPC, blood platelet cell; PB Blast, blast cell count of peripheral blood; BM Blast, blast cell count of bone marrow; NK, abnormal karyotype; WT, wild type; *CSF3R*, colony-stimulating factor 3 receptor;*SETBP1*, SET-binding protein 1; *SRSF2*, serine/arginine-rich splicing factor 2.

**Table 3:** CMML: patient characteristics and correlation with SRSF2 mutation status

variables	*SRSF2* MUT(*n* = 14)	*SRSF2* WT (*n* = 42)	*P*
Sex (female/male)	4/10	15/27	> 0.05
Age (years)	66.7 (25–82)	62.4 (36–94)	> 0.05
WHO subtypes			
CMML-1	10	33	> 0.05
CMML-2	4	9	
FAB subtypes			
CMML-MD	2	10	> 0.05
CMML-MP	12	32	
CPSS			
low risk	2	4	
intermediate-1 risk	7	30	> 0.05
intermediate- 2 risk	5	7	
high risk	0	1	
BM Blast (%)	3.13 ± 1.7	3.04 ± 1.8	> 0.05
PB Blast (%)	3.13 ± 1.7	3.18 ± 1.87	> 0.05
WBC (×10^9^/L)	36.1 ± 17.9	33.0 ± 13.1	> 0.05
HB (g/L)	98.4 ± 16.7	94.4 ± 11.5	> 0.05
BPC (×10^9^/L)	225.6 ± 116.3	222.4 ± 135.8	> 0.05
Mono (×10^9^/L)	4.58 ± 3.1	3.2 ± 1.08	> 0.05
Mono (%)	12.8 ± 3.38	15.1 ± 4.82	> 0.05
*CSF3R* mutation	0/10	4/42	< 0.001
*SETBP1* mutation	2/14	1/42	> 0.05

CMML, chronic myelomonocytic leukemia; WHO, World Health Organization; FAB, French, American and Britain; CMML-MD, CMML-myelodysplastic; CMML-MD, CMML- myeloproliferative; MUT, mutated; WT, wild type; CPSS, CMML Prognostic Scoring System; PB Blast, blast cell count of peripheral blood; BM Blast, blast cell count of bone marrow; WBC, white blood cell; HB, hemoglobin; BPC, blood platelet cell; Mono, monocyte.

Patients with *CSF3R* mutation had higher HB levels (*P* = 0.05) compared to wt *CSF3R* patients. However, no statistically significant difference was identified between mut *CSF3R* and wt *CSF3R* CMML patients in terms of age, gender, WHO category, FAB category, karyotype, other blood cell counts and CPSS risk stratification. In terms of treatment for the investigated patients, we normally used hydroxyurea and Interferon-α for CNL patients, and demethylation therapy, combined chemotherapy, hydroxyurea, immunomodulation therapy and supportive treatment for CMML patients.

### *SRSF2* and *SETBP1* mutations are independent predictors of poor survival for CMML patients

**Figure 3:**
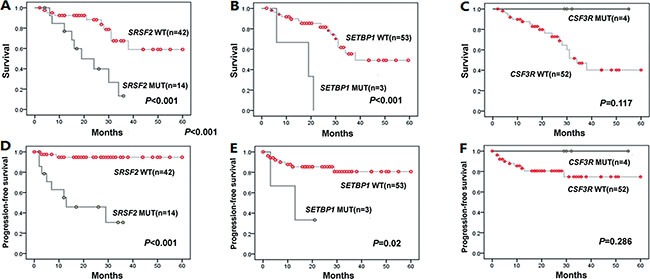
Kaplan-Meier curves for OS, PFS according to genotypes with statistical significance in univariate analysis (**A**–**C**) Overall survival (OS) for *SRSF2*, *SETBP1* and *CSF3R* cases. (**D**–**F**) Progression-free survival (PFS) for *SRSF2*, *SETBP1* and *CSF3R* cases. In univariate analysis, *SRSF2* and *SETBP1* mutations suggested a poor prognosis for OS (*P <* 0.001both) and PFS (*P <* 0.001 and *P* = 0.02, respectively). There was no statistical significance of *CSF3R* mutations in OS and PFS (*P >* 0.05).

We next focused our prognostic analysis on the impact of mutation status in CMML patients. Overall, during a median follow-up period of 20 months (range: 2–60 months), 14(25%) patients died, mainly due to progression to AML(10/14). Both *SRSF2* and *SETBP1* mutated patients showed shorter progression-free survival (PFS) and overall survival (OS) compared with wt *SRSF2* (both *P <* 0.001) and wt *SETBP1* (*P* = 0.02 and *P <* 0.001) patients. In addition, we observed that patients with *CSF3R* mutations had longer PFS and OS compared to wt *CSF3R* patients. However, the difference was not statistically significant (*P >* 0.05) (Figure 3).

In univariate analysis, *SRSF2* mutations (HR, 4.74; 95% CI, 1.811 to 12.42; *P* = 0.002 for OS; HR, 15.39; 95% CI, 3.25 to 72.81; *P* = 0.001 for PFS, respectively), *SETBP1* mutations (HR, 8.25; 95% CI, 2.12 to 32.07; *P* = 0.002 for OS; HR, 5.29; 95% CI, 1.10 to 25.51; *P* = 0.038 for PFS, respectively), higher BM blast counts (HR, 1.18; 95% CI, 1.05 to 1.32; *P* = 0.006 for OS; HR, 1.14; 95% CI, 1.01 to 1.29; *P* = 0.029 for PFS, respectively), higher PB blast counts (HR, 1.14; 95% CI, 1.02 to 1.27; *P* = 0.02 for OS; HR, 1.13; 95% CI, 1.01 to 1.26; *P* = 0.028 for PFS, respectively), higher peripheral blood mononuclear cells (PBMC) (HR, 1.13; 95% CI, 1.02 to 1.25; *P* = 0.015 for OS) and older age (HR, 1.04; 95% CI, 1.01 to 1.07; *P* = 0.017 for OS) suggested a poor prognosis, while HB (HR, 0.941; 95% CI, 0.941 to 0.998; *P* = 0.038 for PFS) was associated with a favorable PFS. Overall, there was no statistically significant difference in OS and PFS based on sex, WHO-subtype, FAB-subtype, WBC, BPC and cytogenetics (*P >* 0.05 for all comparisons) (Table 4).

**Table 4:** The univariate and multivariate Cox regression analysis of survival in CMML patients

Variables	OS						PFS					
	univariate analysis			multivariate analysis			univariate analysis			multivariate analysis		
	*P*	HR	95%CI	*P*	HR	95%CI	*P*	HR	95%CI	*P*	HR	95%CI
*SRSF2* mutations	0.002	4.74	1.811–12.42	0.028	3.31	1.14–9.61	0.001	15.39	3.25–72.81	0.001	15.43	3.041–78.312
*SETBP1* mutations	0.002	8.25	2.12–32.07	0.034	9.49	1.18–76.13	0.038	5.29	1.10–25.51	NS	NS	NS
BM Blast	0.006	1.18	1.05–1.32	NS	NS	NS	0.029	1.14	1.01–1.29	NS	NS	NS
PB Blast	0.02	1.14	1.02–1.27	NS	NS	NS	0.028	1.13	1.01–1.26	NS	NS	NS
Mono	0.015	1.13	1.02–1.25	NS	NS	NS	NS	NS	NS	NS	NS	NS
Age	0.017	1.04	1.01–1.07	NS	NS	NS	NS	NS	NS	NS	NS	NS
HB	NS	NS	NS	NS	NS	NS	0.038	0.941	0.941–0.998	NS	NS	NS
Sex	NS	NS	NS	NS	NS	NS	NS	NS	NS	NS	NS	NS
WHO-subtype	NS	NS	NS	NS	NS	NS	NS	NS	NS	NS	NS	NS
FAB-subtype	NS	NS	NS	NS	NS	NS	NS	NS	NS	NS	NS	NS
WBC	NS	NS	NS	NS	NS	NS	NS	NS	NS	NS	NS	NS
BPC	NS	NS	NS	NS	NS	NS	NS	NS	NS	NS	NS	NS
Mono %	NS	NS	NS	NS	NS	NS	NS	NS	NS	NS	NS	NS
Cytogenetics	NS	NS	NS	NS	NS	NS	NS	NS	NS	NS	NS	NS

CMML, chronic myelomonocytic leukemia; OS, overall survival; PFS, progression-free survival;BM Blast, blast cell count of bone marrow; PB Blast, blast cell count of peripheral blood; WBC, white blood cell; HB, hemoglobin; BPC, blood platelet cell; Mono, monocyte; HR, hazard ratio; CI, confidence interval.

In multivariate analysis, *SRSF2* mutations were found to be independent poor predictors for OS (HR, 3.307; 95% CI, 1.137 to 9.614; *P* = 0.028) and PFS (HR, 15.431; 95% CI, 3.041to 78.312; *P* = 0.001). Likewise, *SETBP1* mutations were independent predictors of poor OS (HR, 9.492; 95% CI, 1.183to 76.128; *P* = 0.034) (Table 4).

## DISCUSSION

Next generation sequencing studies have revealed a large number of mutations in genes such as TET2, CBL, AXSL1, RUNX1, EZH2, RAS, JAK2, IDH1/IDH2, NPM1 and spliceosome mutations in CMML [18] as well as *CSF3R*, *SETBP1* mutations in CNL and aCML [19]. In this study, we investigated the frequencies, diagnostic significance and clinical outcome of *CSF3R*, *SETBP1* and *SRSF2* gene mutations in CNL and CMML patients.

Our study show that 80% of CNL patients carried *CSF3R* mutations, with the substitution mutation of *CSF3R* T618I occurring exclusively in WHO-defined CNL. However, *CSF3R* mutations were not seen in MGUS-CNL, MDS, CEL or healthy donors. Pardanani et al. have identified *CSF3R* T618I in 83% of CNL patients, which did not affect survival [9]. In contrast, patients with *SETBP1* mutations (33% in CNL) experienced shortened survival [9]. Because of the limited number of mutated cases, we only described the clinical characteristics of CNL patients without analyzing the prognostic status of these patients (Table 2).

We identified *CSF3R* mutations in 7.1% of CMML patients. Interestingly, all of the *CSF3R* mutations detected in CMML patients were *CSF3R* P733T mutations. *CSF3R* P733T mutation represents a novel *CSF3R* point mutation not previously reported. In addition, none of the 4 *CSF3R* P733T mutated patients were detected with *SRSF2* mutations, suggesting that *CSF3R* P733T mutations and *SRSF2* mutations are mutually exclusive in CMML patients. Therefore, the *CSF3R* P733T mutation may be of diagnostic value in CMML, especially for wt *SRSF2* CMML patients. In univariate analysis, we observed that CMML patients with *CSF3R* mutations had longer PFS and OS compared to wt *CSF3R* patients. However, the difference was not statistically significant. The lack of significance may be due to the small sample size in the *CSF3R* mutant group which resulted in low statistical power. In contrast, Kosmider and collegues identified *CSF3R* mutations in 3% CMML patients and showed that *CSF3R* mutations were associated with an reduced OS and AML-free survival in univariate analysis [8]. Additionally, Maxson et al. showed that *CSF3R* mutations were divided into membrane proximal mutations and truncation mutations based on the distribution within two distinct regions of *CSF3R* [2]. Truncation mutations in *CSF3R* activates downstream signaling mediators-SRC family kinases (SFKs) and TNK2 and lead to increased sensitivity to Dasatinib treatment [2]. Membrane proximal mutations, on the other hand, showed preferential activation of JAK signaling pathway and susceptible to JAK kinase inhibitors such as Ruxolitinib [2]. Fundamental differences in mutations types might therefore indicate differences in prognosis of CMML patients, which deserves further attention.

Here, we demonstrate *SETBP1* mutations present in 5.3% of CMML patients. In univariate analysis, *SETBP1* mutations was an independent adverse predicting factor for OS (*P* = 0.002) and PFS (*P* = 0.038). Similar results were obtained for OS in multivariate analysis. These data are consistent with multiple previous studies [15, 16, 19–21], which have shown that *SETBP1* mutations occur in 4–7% CMML patients and are indicative of decreased OS and AML-free survival [22].

Notably, *SRSF2* mutations were detected in 25% CMML patients in our study but exclusive of mutations in *CSF3R*. Our multivariate analysis showed *SRSF2* mutation as an independent poor predictor for OS (*P* = 0.028) and PFS (*P* = 0.001), in line with previous data from Itzykson et al. and Makishima et al. [11, 23].

In conclusion, we report that the majority of CNL patients studied here carried oncogenic *CSF3R* mutations and that the *CSF3R* T618I mutation appears to be a diagnostic marker with good specificity and sensitivity for CNL. By contrast, *CSF3R* P733T mutations detected in CMML patients were completely different from *CSF3R* mutation types described in patients with CNL, SCN and hereditary neutrophilia, which may be a potential diagnostic marker, particularly for wt *SRSF2* CMML patients. In addition, mutations in *SRSF2* were commonly found in CMML patients, and represents a poor prognostic marker for CMML. Mutations in *SETBP1*, by contrast, were found in CNL, CMML but also other hematological malignancies, making it a rather poor isolated prognostic marker for hematological diseases. As patients with different gene mutations may have different clinical response to treatment, mutation-based personalized targeted therapy should be considered in future studies.

## MATERIALS AND METHODS

### Study population and definitions

A total of 10 CNL patients and 56 CMML patients were included in the study. 43 were male and 23 were female, at a median age of 64 (range: 24–94) years. Of the 56 CMML patients, 47 were diagnosed with CMML-1 and 9 were diagnosed with CMML-2. Of the 10 CNL patients, 1 was diagnosed with monoclonal gammopathy of undetermined significance associated with chronic neutrophilic leukemia (MGUS-CNL). 20 MDS patients, 10 chronic eosinophilic leukemia (CEL) patients and 20 healthy donors were included as controls. The diagnosis, classification and AML transformation of CMML, CNL, MDS and CEL were based on WHO 2008 criteria [1].

### *CSF3R*, *SRSF2* and *SETBP1* mutation analysis

We obtained DNA specimens from bone marrow mononuclear cells or peripheral-blood granulocytes from the patients, and constitutional DNA samples from matched buccal swabs as described previously [9]. *CSF3R* exons 14–17, *SETBP1* exon 4 and *SRSF2* exon 1 were amplified. Primers used were: *CSF3R* exon 14 F: CCACGGA GGCAGCTTTAC; *CSF3R* exon 14 R: AAATCAGCATC CTTTGGG TG. *CSF3R* exon 15 F: TGACTTTGAATCC CCTGGTC; *CSF3R* exon 15 R: TGAGGTTCCCTGT GGGTG; *CSF3R* exon 16 F: AAAATGGAAAGATC GGAG GG; *CSF3R* exon 16 R: CTTGGCTTCAGAAG GTGTCC; *CSF3R* exon 17 F: CTGTCACTTCCGGC AACAT; *CSF3R* exon 17 R: TGGCCCAAAGACAC AG TCGT; *SETBP1* exon 4 F: ACCTGGAAGCTGTCTCC ACCCA; *SETBP1* exon 4 R: CGGTGGCCATGCCGG TTCTT; *SRSF2* exon 1 F: CTGTCACTTCCGGC AACAT; *SRSF2* exon 1 R: TGGCCCAAAGA CACAGTCGT.

Independent validations of the detected variants were conducted using Sanger sequencing. If nonsynonymous sequence changes were detected, we determined whether the sequence variant had previously been reported as a SNP by searching in the Single Nucleotide Polymorphism database (https://www.ncbi.nlm.nih.gov/projects/SNP/). The previously unreported variants were resequenced with the matched buccal swabs samples DNA from the same patients. The somatic mutation was confirmed when it associated with the bone marrow or peripheral-blood sample DNA and not the matched buccal swabs DNA.

### Study of clinical features

Patient data was collected at the first diagnosis. The median follow-up period was 20 months (range: 2–60). CMML patients were divided into high, intermediate-1, intermediate-2 and low risk group according to the CMML Prognostic Scoring System (CPSS) [24].

### Statistical analysis

The statistical analysis of data was done by using Excel and SPSS (statistical package for social science) version 17.0. Statistical analysis was performed by comparison between groups using Kruskal-Wallis test regarding quantitative nonparametric data and chi-square test regarding qualitative data. The overall survival analysis was done by Kaplan–Meier curve. Multivariate analysis of survival was carried out by Cox regression. All *P*-values < 0.05 (two-tailed) were considered statistically significant.
